# Evaluation of Nurses' Knowledge Levels of Diabetic Foot Care Management

**DOI:** 10.1155/2018/8549567

**Published:** 2018-07-02

**Authors:** Zahide Kaya, Anita Karaca

**Affiliations:** ^1^Uskudar State Hospital, Turkey; ^2^Florence Nightingale Hospital School of Nursing, Istanbul Bilim University, Turkey

## Abstract

**Background:**

Nurses, important members of the diabetes treatment team, have an essential role in the prevention of diabetic foot problems and in the care and education of patients at risk of diabetic foot problems.

**Objective:**

The study evaluated the knowledge levels of nurses regarding diabetic foot care management and determined influencing factors.

**Methods:**

This was a cross-sectional, descriptive study. The research sample comprised 435 nurses who worked in a private hospital. The research data were collected using the “Nurse Information Form” and “Nurses' Knowledge Level Form on Diabetic Foot Management”.

**Results:**

It was found that 66% of the nurses did not receive training in diabetic foot care, 80.9% did not educate patients with diabetic foot problems, and 77.5% did not perform foot examinations on diabetic patients.

**Conclusion:**

Nurses' knowledge level scores regarding diabetic foot management are adequate, but this knowledge is not used during patient care. In order to facilitate nurses' involvement in diabetic foot management, theoretical and practical training programs should be organized and nurses should be encouraged to participate in these programs.

## 1. Introduction

The diabetic foot is a serious complication of diabetes with high mortality, morbidity, and cost of treatment, which can be prevented by patient education and early diagnosis-treatment [1–4]. Diabetic foot problems are a frequent cause of hospital admissions for patients with diabetes and comprise the main factor determining the quality of life of diabetic patients [5, 6]. Diabetic foot problems not only cause the patient to lose work/income, hinder their educational pursuits, and damage social relations, but also cause harm to patients psychologically and to their environments indirectly [7].

Risk factors must be known and monitored to prevent diabetic foot complications. The most important risk factors for foot ulceration include peripheral neuropathy, peripheral vascular disease, foot deformity, previous foot ulceration, and amputation of the foot or leg [8–10]. In addition, recurrent chronic abrasions, minor abrasions, bullae, various irritations, verrucas and calluses, improper cutting of toenails, fungal infection, poor foot hygiene, inappropriate footwear use, and bad metabolic control are the most common causes of foot ulcer formation in patients [6, 8, 11]. Because of these changes, feet are easier to traumatize and wounds heal slowly, which increases the risk of infection. In order to control these risk factors, all patients with diabetes should be examined at least once a year for potential foot problems, and those with risk factor(s) should be examined every 3-6 months [3, 4]. Diagnosis of the foot at risk, regular examination of the foot at risk, education of patients, family, and health workers, management of nonulcerative pathologies, and management of the diabetic ulcer are the main elements of diabetic foot management [12–14]. In addition, other risk factors such as hypertension, alcohol, smoking, hyperlipidemia, obesity, and visual impairment should be addressed in patients [11, 15, 16].

Foot screening and assessment to identify the high-risk foot are aimed at preventing the serious complications of ulceration and amputation. The protective sensory feelings, foot structure and biomechanics, vascular structure, and skin integrity should be assessed during diabetic foot examinations [6, 17]. When examining the foot at risk, vasculature (limping, pain at rest, and palpation of the foot pulse), skin (callus, color, heat, edema, texture, and foot ulcer), and bone/joint condition (claw toes, hammer toes, hallux valgus, hallux limitus, equinus, amputation, Charcot deformity, drop foot, and joint limitation) are evaluated [13, 14]. Diabetic individuals should be questioned during neurological evaluations for neuropathy findings. A 10-g Semmes-Weinstein monofilament set, which is an inexpensive, painless, and easy method, is used to evaluate the loss of protective sensation in the foot [16, 18–20]. A 10 g pressure is applied to certain points in the plantar and dorsal areas of the patient foot. If sensory loss is detected during patient evaluations using this filament, the foot is in danger and the protective sense has disappeared [11, 21]. The diagnosis of the foot at risk is confirmed with a vibration test (using a 128-Hz tuning fork or a biothesiometer), pinprick sensation, or ankle reflexes [17, 18, 20, 22]. Patients with neuropathy, especially those with foot deformities or previous ulcer/amputation history, should be careful when choosing shoes. A patient at risk should be encouraged to wear therapeutic shoes that reduce plantar pressure while walking to prevent recurrent plantar foot ulcers [4, 13]. The data obtained during the foot examination determine which risk category patients belong to for diabetic foot problems [11, 19]. These categories are designed to facilitate referral to, and subsequent therapy by, a specialty clinician or team and determine follow-up frequency. A high-risk category is associated with an increased risk for ulceration, hospitalization, and amputation [11, 22, 23].

The diabetic foot needs a multidisciplinary team approach because it requires long-term treatment utilizing many areas of expertise [12, 15, 24, 25]. Multidisciplinary team work can reduce foot ulcer and amputation rates, decrease healthcare costs, and lead to better quality of life for patients with diabetic foot ulcer risk [26, 27]. The members of the diabetic foot care team usually consist of a general practitioner, nurse, educator, orthotist, podiatrist, vascular surgeon, infection disease specialist, dermatologist, endocrinologist, dietitian, and orthopedic surgeon [17, 22, 25]. Although all team members should educate the patient, the nurse and podiatrist are often the primary sources of patient information [27].

Lack of proper education and awareness of regular foot care play a contributory role in the causation of foot problems [28–30]. A specific education course for foot and wound care decreases the rate of foot ulcers and amputations, and existing guidelines state the need for patient education as a prerequisite to prevent ulceration [31, 32]. In fact, educating patients on foot self-management is considered the cornerstone to prevent diabetic foot ulcers. The goals of training are to motivate the patient and create adequate skills to maximize the use of preventive methods [6]. However, nurses are the primary point of contact for patients and are seen as a source of information by patients. In order for nurses to fulfill this role, they must have knowledge regarding diabetic foot care management and convey this knowledge to the patient [33–37]. Therefore, we assessed the knowledge level of nurses of diabetic foot care and their use of this knowledge in patient care.

*Objective*. The aim of this study was to evaluate the knowledge levels of nurses of diabetic foot care management and to determine influencing factors.

*Research Questions*

- What is the level of nurses' knowledge about management of diabetic foot care?
- Is there a difference between the levels of nurses' knowledge about management of diabetic foot care according to their sociodemographic, occupational and diabetic foot management characteristics?

## 2. Methods

### 2.1. Design

This was a descriptive cross-sectional study.

### 2.2. Sample and Setting

This study was conducted in a private hospital in Istanbul with joint commission international accreditation. It is one of the largest groups of hospitals in the country and ranks among the best hospitals in Turkey in the fields of cardiology, cardiovascular surgery, and organ, tissue, and cell transplants. In addition, it serves as a training hospital for many specialties by combining its academic activities with health services. This study was carried out in three hospitals including one application and research hospital affiliated with a foundation university and two private hospitals.

The research population was 540 nurses working in the hospitals. A random sampling method was used to select the study sample. The study sample consisted of 435 nurses (response rate: 80.5%) who were working and agreed to participate in the study between September 01 and December 01, 2016. Nurses who did not agree to participate in the study or who could not be reached for various reasons (annual leave, vacation, maternity leave, etc.) were not included in the survey.

The nurses who agreed to participate in the research were asked to answer questionnaire forms by the researcher. The test was self-administered and took 15-20 minutes. The completed questionnaire forms were collected by the researcher.

### 2.3. Instruments

Data collection tools consisted of the “Nurse Information Form” and “Nurses' Knowledge Level Form on Diabetic Foot Management”.

*Nurse Information Form*. The “Nurse Information Form” consisted of 2 sections with 15 questions in total. There were 8 questions about nurses' sociodemographic and professional characteristics and 7 questions related to nurses' diabetic foot management care.

*Nurses' Knowledge Level Form on Diabetic Foot Management*. The “Nurses' Knowledge Level Form on Diabetic Foot Management” was used to evaluate nurses' knowledge level about diabetic foot management. There is no valid and reliable measurement tool in our country to measure the level of knowledge of nurses regarding diabetic foot care management. Therefore, a questionnaire form was prepared. This form can be used as a guiding resource in developing valid and reliable measurement tools in the future to measure knowledge about diabetic foot management in Turkey. The test was developed for this study after the related literature was consulted [3, 4, 13, 36]. The “Nurses' Knowledge Level Form on Diabetic Foot Management” consisted of 68 true/false questions divided into 4 sections: “Risk Factors” (16 questions), “Foot Examination” (10 questions), “Foot Complications” (32 questions), and “Footwear Selection” (10 questions). Each correct answer was encoded as “1” and each incorrect answer was encoded as “0”. The lowest score possible was “0” and the highest possible score was “68”. The higher the total score, the higher the knowledge level of diabetic foot management. The opinions of five experts in nursing fundamentals (1), internal diseases nursing (3), and surgical diseases nursing (2) were obtained to assess the items in terms of statement and content/scope validity in the survey form. The experts assessed the scale items for their fitness for the purpose. They scored each item from 1 to 3: 1=not suitable to the content and not understandable, 2=can be suitable when provided with amendment, 3=suitable to the content and clearly expressed. The experts' mean score for each item was 2 or higher. The questionnaire was also administered to a group of 15 nurses prior to use in the study to assess whether the questions were clear and understandable. Some minor corrections were made on the questionnaire form in line with the suggestions received from the preliminary application of the form, and the questionnaire form was finalized.

In this study, the reliability coefficient of the Nurses' Knowledge Level Form on Diabetic Foot Management (Cronbach's *α*) was found to be 0.82 for the “Risk Factors” subscale, 0.63 for the “Foot Examination” subscale, 0.82 for the “Foot Complications” subscale, and 0.79 for the “Footwear Selection” subscale. The reliability coefficient for the entire form was found to be 0.90, a high reliability value as a result of validity and reliability test performed for the whole scale.

### 2.4. Ethical Considerations

Before starting the study, written consent was obtained from the hospitals where the research was conducted with the approval of the Clinical Research Ethics Committee (Decision No: 16.08.2016/53-16). In addition, before collecting the data, the nurses were asked to sign an “Informed Volunteer Consent Form” after they were informed about the purpose and methodology of the study.

### 2.5. Statistical Analyses

The data were analyzed with the program “SPSS for Windows version 15.00”. The sociodemographic and diabetic foot management practices of nurses were determined as independent variables, and their knowledge level scores relating to diabetic foot management were determined as dependent variables. Descriptive statistics (means, standard deviations, frequencies, and percentages) were calculated for demographic variables. The suitability of the data for normal distribution was tested using the Single Sample Kolmogorov Smirnov test and parametric tests were used in the advanced analysis because the significance values were greater than 0.05. Associations between background factors and the foot care knowledge test were analyzed using a t-test for paired group comparisons and one-way ANOVA for more than two-group comparisons. The relationship between variables was examined by Pearson correlation analysis. Internal consistency of the scale was tested using Cronbach's alpha.

## 3. Results

### 3.1. Sociodemographic and Professional Characteristics of Nurses

The average age of the nurses participating in the survey was 26.37±4.97. More than half of the nurses were female (76.8%), were single (72.6%), and had an undergraduate level of education (61.1%). When the distribution of the nurses according to the unit where they were working was examined, 21.1% were working in the intensive care unit and 66.7% were service nurses. The mean duration of occupational time was 61.22±57.40 months (Table 1).

### 3.2. Characteristics of Nurses on Diabetic Foot Management Care

One-third of the nurses (34%) were trained in diabetic foot care and 29% received training related to the diabetic foot within the curriculum of nursing education. However, 80.9% of the nurses did not educate patients with diabetic foot risk or problems. The nurses provided the most patient education regarding blood sugar control (18.6%). In addition, 77.5% of the nurses did not perform a diabetic foot examination for diabetic patients and 42.8% stated they needed training in diabetic foot care, primarily in risk factors of the diabetic foot and its etiology (36.1%) (Table 2).

### 3.3. Distribution of Nurses' Knowledge Level Form on Diabetic Foot Management Scores

The average score on the Nurses' Knowledge Level Form on Diabetic Foot Management was 58.67±5.94. The distribution of knowledge level scores regarding diabetic foot management of the nurses in the study is given in Table 3. The highest score was in the “Foot Complications” section (26.47±2.64), whereas the lowest score was in the “Footwear Selection” section (8.24±1.64).

The participating nurses correctly answered the items regarding “poor glycemic control” (98.4%) and “color control is made” (99.8%). The correct answers were given to “feet should be checked every day by the patient or a relative by eye, hand, and mirror” (98.9) and “if there is a deformity in the foot, a doctor should be consulted for the appropriate treatment or orthopedic shoes” (96.8%). Items nurses answered incorrectly on the Nurses' Knowledge Level Form on Diabetic Foot Management were “presence of foot callus” (21.8%) in “Risk Factors”, “muscle functions are assessed” (15.6%) in “Foot Examination”, “callus and skin stiffness should be thinned with a pumice stone” (20.0%) in “Foot Complications”, and “shoes should be painted frequently” (32.2%) in “Footwear Selection” (Table 4).

### 3.4. Nurses' Significant Sociodemographic, Professional, and Diabetic Foot Management Care Characteristics Compared with the Knowledge Form on Diabetic Foot Management Scores

Significant sociodemographic, professional, and diabetic foot management characteristics of nurses in the study were compared to nurses' knowledge level scores. There was no statistically significant correlation between knowledge level scores and nurses' gender, marital status, duration of work in the unit, educating patients with diabetic foot risk or problems, and performing patients' foot examinations (p>0.05).

According to the age group of the nurses, the difference between the score on the “Foot Complications” dimension and the “Total Score” was statistically significant (p<0.05). The score of “Foot Complications” and “Total Score” (28.63±1.77 and 62.88±3.56, respectively) of the nurses aged 40 and higher were significantly higher than the scores of the nurses aged 18-19 years (24.88±2.85 and 56.38±4.53, respectively).

The difference between the education status of the nurses in the sample and the score of the “Footwear Selection” dimension was statistically significant (p<0.05). The scores of the “Footwear Selection” dimension (8.36±1.57) of the nurses with an undergraduate education level were significantly higher than the scores of the nurses with vocational high school health education (7.78±2.05).

According to the occupational duration of the study group, there was a significant, positive, and very low relationship between “Risk Factors”, “Foot Complications”, and “Total Points” (r_p_=0.116, r_p_=0.094, and r_p_=0.102, respectively). On the other hand, there was no statistically significant relationship between nurses' occupational duration and the scores on “Foot Examination” and “Footwear Selection” (p>0.05).

There was a statistically significant difference between nurses' status of receiving training about diabetic foot care and all the scores except “Foot Examination” (p<0.05). The scores for “Risk Factors”, “Foot Complications”, “Footwear Selection”, and “Total Scores” of the nurses trained in diabetic foot care are significantly higher than the scores of nurses who are not trained in diabetic foot care (Table 5).

## 4. Discussion

Nurses on the healthcare team have contact with patients for 24 hours and thus play an important role in educating patients [20, 38]. Nurses can improve the quality of life of a diabetic individual by assisting in the preparation and implementation of education programs that help patients develop self-care behaviors related to diabetic foot care. In addition, they can prevent or delay formation of diabetic foot problems by identifying risk groups in the community [13, 27]. Therefore, nurses' knowledge levels must be assessed periodically using validity and reliability tools. Theoretical and practical deficiencies can be revised, false information can be corrected, and nurses' knowledge and skills can be improved through obtaining evidence-based data regarding their knowledge, skills, and practices.

The variables to be measured by a good measurement tool must fit for purpose, include cognitive scales related to the subject, and have information to obtain correct data. The survey form that was used in this study was prepared based on the researchers' previous experience and information from previous studies. In addition, it attempted to address all the factors that affect the development of diabetic foot after an extensive review of the literature scanning. The form not only includes practices about diabetic foot care, but also statements regarding factors that play a key role in diabetic foot development, choice of suitable shoes, and foot examination. Moreover, in the process of designing the items, a great deal of attention was paid for the items not to have more than one statement or opinion and to be clear and understandable. Experts were consulted to assess the measurement tool and to obtain a more reliable and understandable form. The scale had high reliability too. This survey is thought to be helpful for future studies to be carried out on this subject.

The knowledge levels of nurses of diabetic foot care management and influencing factors were examined in this study. The knowledge level of nurses was high, but they did not provide patients with adequate education on this subject or examine the foot. This suggests that nurses' awareness of diabetic foot management should be increased and that they should apply their theoretical information in the clinical field.

### 4.1. Nurses' Characteristics Related to Diabetic Foot Management

The most important prevention of diabetic foot problems is the repeated education of all diabetic patients at every health visit [31]. In our study, 80.9% of the nurses did not train patients at risk of or with diabetic foot problems. Although the nurses stated that their knowledge level about diabetic foot management was adequate and there was no need for additional training, practices in this area were inadequate. Another study [39] indicated that the diabetic foot constitutes a heavy patient burden both physically and mentally, but it can be prevented with the correct patient education and regular preventive care and treatment. All health professionals play a role in diabetic foot treatment, but nurses play a significant role because they are in communication with patients for 24 hours. Moreira and Sales [40] stated that it would be best for people with diabetic foot diseases to perform their own care. Nurses should educate the patient and then direct the patient rather than taking control of the care. Similarly, Ren et al. [41] investigated the importance of nursing education in high-risk diabetic foot patients. In the two-year follow-up, the patients specifically educated regarding foot care showed more improvement than the control group. Moreover, the training prevented foot ulcers and reduced amputations. In a separate study [19], only 29.6% of the patients were trained in foot care, with 87% of the education given by doctors and 5.2% given by nurses. In Batkın and Çetinkaya's study [42], 18.4% of the patients were informed about foot care, and doctors (80.7%) were the first health provider from whom they received information. These studies show that nurses do not play an active role in diabetic foot education, perhaps because patients encounter physicians more during their examination or the nurses lack awareness of this issue. In light of these findings, nurses should improve their patient education efforts using the knowledge they already possess.

Nurses need sufficient knowledge and skills in foot care to prevent, diagnose, and care for foot problems. Thus, it is important that nurses' foot care knowledge be supported with practical training [36]. In our study, 34% of the nurses were trained in diabetic foot care and 42.8% stated that they needed training in diabetic foot care. Namwong [43] found that nurses and trainers have inadequate knowledge of diabetic foot care, do not practice it, and have insufficient knowledge to divide patients into groups according to foot risk levels. According to Stolt et al. [36], while the majority of nurses (71%) were theoretically trained in foot care, 17% received both theoretical and applied foot care training and 10% received only applied foot care training. Nurses stated that foot care training in vocational education and their current foot care knowledge are insufficient. In a related study, Aalaa et al. [27] investigated the role of nurses in the prevention and treatment of the diabetic foot and reported that patients see nurses as teachers in matters such as the prevention of diabetic foot problems, preventive care of the foot, and prevention of foot wounds. In the light of these studies, nurses should have sufficient knowledge and skills about the topic, but nurses generally are not trained in diabetic foot care. In addition, some studies show the theoretical training should be supported with practical training.

Foot examination in the early diagnosis and treatment of diabetic foot problems is important. Proper footwear and regular examination of feet for signs of neuropathy, impaired blood flow, and skin changes can prevent foot ulcers that often lead to gangrene and limb amputation. Active participation of nurses should occur during diabetic foot care and foot examination [6, 31]. In our study, 77.5% of the nurses did not perform a foot examination for diabetic patients. The results of our study are similar to those of Namwong [43]. In Karaca and Enç's study [19], 34.2% of patients had previously undergone foot examinations, but physicians performed all foot examinations and nurses did not play a role. Likewise, Waheida et al. [20] illustrated that no nurses in their study had previous experience with the monofilament examination or tuning fork assessment of the dorsalis pedis, which are important for early detection of diabetic foot problems. Çaparuşağı and Ovayolu [44] reported that early diagnosis and treatment of diabetic foot are important and nurses have great responsibilities in this respect. These studies showed that nurses should play an active role in foot examinations, but they are not effective in this respect. This may be because nurses do not have enough knowledge about foot examinations or they do not have enough time to do foot examinations. However, it is possible for nurses to examine patients' feet quickly using the standardized forms developed for diabetic foot evaluation after sufficient training.

Training programs about foot care improve nurses' knowledge and practical application of screening tests, which subsequently improve patient outcomes [20]. In our study, when we looked at the status of whether nurses received training in diabetic foot care, those who did had higher knowledge levels of risk factors, foot complications, footwear selection, and general diabetic foot management than those who did not. Similarly, in a study by Shiu and Wong [2], knowledge scores of nurses trained in diabetic foot care were higher than those who were not. In Aydoğan's study [45] on the evaluation of nurses' knowledge level related to diabetes, the group of nurses who received in-service training had a higher knowledge level of diabetes. In a related work, Stolt et al. [36] found that nurses participating in continuing education programs including theoretical and practical education had higher knowledge levels related to foot care. According to Waheida et al. [20], there are significant differences and improvements between nurses assessed before implementation, at implementation, and one month after implementation of a diabetes educational program. For this reason, healthcare organizations must develop clinical expertise on the diabetic foot by implementing diabetic foot assessment and screening into routine assessments and education.

### 4.2. Knowledge Levels of Nurses of Diabetic Foot Management

When we examined the level of knowledge about nurses' diabetic foot management in our study, the knowledge level score was rather high (86.3%). Although nurses' knowledge level of diabetic foot management was sufficient, they did not pay the necessary attention to the education of patients. Nurses who are responsible for diabetic patients should see patients and their relatives as a whole and provide their care and education by predicting the problems that may arise in patients' feet.

In a study conducted by Stolt et al. [36], the majority of nurses had insufficient knowledge about foot care issues. While the highest knowledge scores of nurses regarding foot care were in the subjects of skin and nail care and footwear features in our study, the lowest scores were in identifying deformities in the foot structure and foot care. Shiu and Wong [2] obtained scores averaging 41.4 out of 65 points on their information scale related to diabetic foot care. The most frequently wrong answers included using methyl alcohol between the toes as a risk factor (83.1%), the appropriateness of wearing wool stockings on the foot (75.4%), and the use of hibitane antiseptic solution on minor injuries (73.8%). Ren et al. [41] investigated nurses' knowledge level of diabetic foot care management. Although the majority of the nurses did not receive training in diabetic foot care, the nurses had an adequate level of information. Thus, the knowledge level scores of nurses vary. This may be due to educational level of the nurses or may be related to participating in continuing education programs after basic education. Therefore, the knowledge level of diabetic foot management of nurses working in institutions should be evaluated, and missing aspects should be addressed and misconceptions should be corrected.

### 4.3. Study Limitations

The private hospital where this study was conducted is a group hospital. This study was carried out in a total of 3 hospitals, one of which is an application and research hospital affiliated to a foundation university. Therefore, the results of this study can be generalized only to nurses working in private hospitals in Turkey. We suggest that future studies use larger sample groups with different characteristics, such as state hospitals and education-research hospitals. In addition, the knowledge level form does not include all of the knowledge and practices related to diabetic foot care. However, the instrument does include the central areas of diabetic foot management performed by nurses. In this study, information on neurological and vascular evaluation regarding diabetic foot examination is not comprehensive. The questionnaires observed to have unanswered questions during data entry were not included in the study.

## 5. Conclusion and Recommendations

In conclusion, the knowledge level of nurses about diabetic foot management was rather high, but they did not provide patients at risk of diabetic foot problems with preventive education on foot care or perform foot examinations. In addition, nurses who have worked for a long time in the profession and/or have been trained in diabetic foot care have higher knowledge level scores than those who have not. Nurses in need of training for diabetic foot care were most deficient in diabetic foot risk factors and etiology.

Patient education plays an important role in prevention of diabetic foot problems. Therefore, nurses should take part in the preparation and implementation of training programs that improve self-care behaviors of patients and their quality of life. For this, theoretical and practical in-service training programs on diabetic foot management should be planned to address the training needs of nurses. Combining theory and practice in training programs not only increases nurses' knowledge, but also improves their skills in diabetic foot care. Nurses should be encouraged to participate regularly in these programs or other scientific activities such as courses, seminars, and symposiums, and they should follow professional publications related to the subject. Finally, nurses should be encouraged to use the information they have acquired for the education of diabetic patients.

In particular, the training in diabetic foot management given to nurses can be organized as a separate training program instead of being given in general diabetes education programs. The demonstration method together with oral presentation can be used during the training on foot examination, and the information, attitudes, and behaviors of nurses can be evaluated after the training. In this way, the missing or misunderstood information can be corrected. Thus, nurses active participation in diabetic foot care and foot examinations can be achieved by increasing their awareness of foot problems and formation of diabetic foot ulcers. In addition, undergraduate and postgraduate nursing education curricula for training expert nurses in diabetic foot area can be strengthened with respect to this topic, and practices for foot examinations may be included as a part of general clinical education. Thus, providing patients with education and care by specialized nurses trained in the field of the diabetic foot rather than general nurses may be more effective in preventing diabetic foot problems and reducing amputation. Therefore, the need to acquire sufficient knowledge of foot care can be satisfied and nurses would have the ability to update their knowledge of evidenced-based foot care applications.

## Figures and Tables

**Table 1:** Distribution of nurses according to sociodemographic characteristics.

**Characteristics**	**Category**	**n**	%
Age	Average: 26.37 ± 4.974 (Range: 18-44)		
Gender	Female	334	76.8
	Male	101	23.2
Marital status	Married	119	27.4
	Single	316	72.6
Educational Background	Vocational high school of health	98	22.5
	Associate's degree	48	11.0
	Bachelor's degree	266	61.1
	Graduate degree	23	5.3
Unit	Internal medicine	75	17.2
	Surgery	75	17.2
	Intensive care	92	21.1
	Emergency department	28	6.4
	Operating room	61	14.0
	Polyclinic	32	7.4
	Administration	7	1.6
	Obstetrics/neonatal	24	5.5
	Mixed service	41	9.4
Occupational working time (months)	Average: 61.22±57.396 (range: 0-288)		
Unit working time (months)	Average: 42.05±39.917 (range: 0-240)		
Position	Supervisor	27	6.2
	Service nurse	290	66.7
	Intensive care nurse	86	19.8
	Training nurse	1	0.2
	Executive nurse	7	1.6
	Polyclinic nurse	24	5.5

**Table 2:** Distribution of nurses according to characteristics related to diabetic foot management care.

**Characteristics**	**Category**	** n**	%
Have you received any training on diabetic foot care?	Yes	148	34.0
	No	287	66.0
Where did you get this training on diabetic foot care?*∗*	Within the curriculum of nursing education.	126	29.0
	Within an in-service training program.	40	9.2
	I attended courses, seminars, and symposium programs related to the subject.	9	2.1
	Other	3	0.6
Do you educate patients with diabetic foot risk or problems?	Yes	83	19.1
	No	352	80.9
Which of the following topics do you teach?*∗*	Blood sugar control	81	18.6
	Foot examination	51	11.7
	Foot care	79	18.2
	Footwear selection	51	11.7
	Amputation	21	4.8
Do you perform foot examinations for diabetic patients in your unit?	Yes	98	22.5
	No	337	77.5
Do you think you need training in diabetic foot care?	Yes	186	42.8
	No	239	54.9
What training do you need in diabetic foot care?*∗*	Diabetic foot risk factors and etiology	157	36.1
	Foot examination	111	25.5
	Initiatives to prevent diabetic foot	154	35.4
	Footwear selection	89	20.5

*∗* means more than one option can be marked.

**Table 3:** Distribution of nurses' knowledge level form scores related to diabetic foot management.

**Score**	**Potential Distribution**	**M ± SD**	**Min**	**Max**
(F1) Risk Factors	0-16	14.49 ± 2.54	4	16
(F2) Foot Examination	0-10	9.46 ± 1.15	2	10
(F3) Foot Complications	0-32	26.47 ± 2.64	11	32
(F4) Footwear Selection	0-10	8.24 ± 1.64	0	10
Total Score	0-68	58.67 ± 5.94	34	67

**Table 4:** Nurses' Knowledge Level Form on Diabetic Foot Management.

**RISK FACTORS**	**True**		**False**	
	**n**	%	**n**	%

(1) Poor glycemic control	**428**	98.4	7	1.6
(2) Presence of sense of chill, pain, burning, tingling, and tenderness in foot	**408**	93.8	27	6.2
(3) Neuropathic foot (loss of sensory-motor function)	**417**	95.9	18	4.1
(4) Peripheral vascular disease	**369**	84.8	66	15.2
(5) Inadequate foot care and lack of hygiene	**409**	94.0	26	6.0
(6) Presence of foot edema	**379**	87.1	56	12.9
(7) Presence of foot callus	**340**	78.2	95	21.8
(8) Dry and cracked foot skin	**362**	83.2	73	16.8
(9) Those with diabetic foot history or diabetic ulcers in opposite extremity	**423**	97.2	12	2.8
(10) Infection (redness, tenderness, and temperature increase are present in foot)	**423**	97.2	12	2.8
(11) Traumas (barefoot walking, bad shoes, accident, foreign body in shoes)	**387**	89.0	48	11.0
(12) Foot deformity (mallet toes, claw toes, hallux valgus, amputation, Charcot deformity, low foot, etc.)	**377**	86.7	58	13.3
(13) Smoking	**404**	92.9	31	7.1
(14) Obesity	**393**	90.3	42	9.7
(15) Age of 65 and over	**377**	86.7	58	13.3
(16) Patients not trained in diabetic foot	**407**	93.6	28	6.4

**FOOT EXAMINATION**				

(1) Foot skin (color change, edema-atrophy, dryness, crack, callus, ulcer, etc.) is evaluated.	**428**	98.4	7	1.6
(2) Color control (pale, cyanosis, red) is made.	**434**	99.8	1	0.2
(3) Temperature control (temperature, coldness) is made.	**425**	97.7	10	2.3
(4) Presence of neuropathy in foot (pain, tingling, burning, tenderness, sensory loss) is evaluated.	**426**	97.9	9	2.1
(5) Muscle functions (atrophy due to motor damage in the muscles) are assessed.	**367**	84.4	68	15.6
(6) Circulatory control (foot is pale and cyanosis) is made.	**426**	97.9	9	2.1
(7) Presence of ulcer on foot (temperature increase in foot, redness, edema, and tenderness) is evaluated.	**431**	99.1	4	0.9
(8) Presence of deformity (hammer finger, claw, hallux valgus, amputation, Charcot deformity, low foot, etc.) is evaluated.	**382**	87.8	53	12.2
(9) Toenails (thickening, ingrowth, and length in the nails) are controlled.	**392**	90.1	43	9.9
(10) Shoe suitability is assessed.	**407**	93.6	28	6.4

**APPLICATIONS FOR PREVENTING FOOT COMPLICATIONS**				

(1) Feet should be checked every day by the patient or a relative by eye, hand, and mirror (callus, crack, redness, bulla, open wound, etc.).	**430**	98.9	5	1.1
(2) Feet should be washed with warm water every day.	**414**	95.2	21	4.8
(3) The water temperature used for washing feet should be checked.	**421**	96.8	14	3.2
(4) Feet, especially spaces between toes, should be dried very well after each wash.	**424**	97.5	11	2.5
(5) Moisturizing cream should be applied to feet.	**405**	93.1	30	6.9
(6) Moisturizing cream should be applied to spaces between toes.	113	26.0	**322**	74.0
(7) Toes should be kept dry to protect from fungal growth.	**425**	97.7	10	2.3
(8) Cutting tools and chemicals should not be used to remove calluses or hardened skin areas.	**415**	95.4	20	4.6
(9) Callus and skin stiffness should be thinned with a pumice stone.	**348**	80.0	87	20.0
(10) Exercise in the form of twisting and stretching toes several times a day should be done to prevent foot corn and callus formation.	**383**	88.0	52	12.0
(11) There is no inconvenience to use callus band and plaster	127	29.2	**308**	70.8
(12) Only socks should be worn to warm feet.	**397**	91.3	38	8.7
(13) Direct heat sources (radiators, hot-water bottle, electrical appliances, etc.) should be used to warm feet.	216	49.7	**219**	50.3
(14) Socks should not be torn, wrinkled, or oversized.	**415**	95.4	20	4.6
(15) Socks should be checked for wetness and color darkness.	**416**	95.6	19	4.4
(16) Socks should be changed every day.	**425**	97.7	10	2.3
(17) Rubber socks preventing circulation should not be worn.	**425**	97.7	10	2.3
(18) Wool socks should be worn in winter and mercerized socks should be worn in summer.	**398**	91.5	37	8.5
(19) Walking with bare feet should not occur.	**406**	93.3	29	6.7
(20) Pressure on feet should be removed by not standing for long periods.	**422**	97.0	13	3.0
(21) Legs should not be crossed when sitting on a chair.	**407**	93.6	28	6.4
(22) If there is clawing of toes, massage should not be done to prevent joint stiffness.	83	19.1	**352**	80.9
(23) Toenails should be controlled in terms of thickening, ingrowth, and length.	**420**	96.6	15	3.4
(24) Toenails should be cut flat.	**394**	90.6	41	9.4
(25) Skin around toenails should not be cut.	**419**	96.3	16	3.7
(26) The thickened nails should be cut with a special scissors after they are softened in warm water.	**414**	95.2	21	4.8
(27) Blind patients must never cut their own toes.	**422**	97.0	13	3.0
(28) The nails should be cut round.	190	43.7	**245**	56.3
(29) Any changes to feet and toes (color, temperature, or shape) and signs of infection should be reported to the doctor immediately.	**422**	97.0	13	3.0
(30) Foot exercises should be done every day to help circulation.	**410**	94.3	25	5.7
(31) In case of any foot lesion, only shoes should be replaced to reduce the load on feet.	87	20.0	**348**	80.0
(32) Smoking is strictly forbidden since it will reduce the amount of blood going to feet.	**423**	97.2	12	2.8

**FOOTWARE SELECTION**				

(1) Shoes should fit and grasp feet.	**416**	95.6	19	4.4
(2) Soft-skinned and comfortable shoes should be preferred.	**417**	95.9	18	4.1
(3) Shoes should be checked for foreign bodies such as nail, gravel, etc. before each wear.	**414**	95.2	21	4.8
(4) Shoes should be worn without socks.	198	45.5	**237**	54.5
(5) If shoe insoles are worn off, they should be replaced.	**411**	94.5	24	5.5
(6) Shoes should not lose its exterior protection feature.	**397**	91.3	38	8.7
(7) Shoes should be painted frequently.	**295**	67.8	140	32.2
(8) New shoes should be worn by allowing feet to get used to them.	**405**	93.1	30	6.9
(9) High-heeled shoes tapering forward should be preferred.	211	48.5	**224**	51.5
(10) If there is a deformity in the foot, a doctor should be consulted for proper treatment or orthopedic shoes.	**421**	96.8	14	3.2

Note: the correct answers were indicated by using bold font for “n”.

**Table 5:** Comparison of nurses' diabetic foot care knowledge level scores to their training on diabetic foot care.

	**Training Status**	** N**	** M ± SD **	*** t***	** p**
(F1) Risk Factors	Yes	148	14.86 ± 2.26	2.181	0.030
	No	287	14.30 ± 2.66		
(F2) Foot Examination	Yes	148	9.51 ± 1.01	0.582	0.561
	No	287	9.44 ± 1.18		
(F3) Foot Complications	Yes	148	26.96 ± 2.35	2.779	0.006
	No	287	26.22 ± 2.75		
(F4) Footwear Selection	Yes	148	8.56 ± 0.95	2.935	0.004
	No	287	8.08 ± 1.89		
Total Score	Yes	148	59.89 ± 4.92	3.103	0.002
	No	287	58.04 ± 6.32		

*t*: Independent-samples t-test.

## Data Availability

The data used to support the findings of this study are available from the corresponding author upon request.

## References

[1] Lavery L. A., Wunderlich R. P., Tredwell J. L. (2005). Disease management for the diabetic foot: effectiveness of a diabetic foot prevention program to reduce amputations and hospitalizations. *Diabetes Research and Clinical Practice*.

[2] Shiu A. T.-Y., Wong R. Y.-M. (2011). Diabetes foot care knowledge: A survey of registered nurses. *Journal of Clinical Nursing*.

[3] Bus S. A., van Netten J. J., Lavery L. A. (2016). IWGDF guidance on the prevention of foot ulcers in at-risk patients with diabetes. *Diabetes/Metabolism Research and Reviews*.

[4] van Netten J. J., Price P. E., Lavery L. A. (2016). Prevention of foot ulcers in the at-risk patient with diabetes: a systematic review. *Diabetes/Metabolism Research and Reviews*.

[5] Lipsky B. A., Aragón-Sánchez J., Diggle M. (2016). IWGDF guidance on the diagnosis and management of foot infections in persons with diabetes. *Diabetes/Metabolism Research and Reviews*.

[6] Iraj B., Khorvash F., Ebneshahidi A., Askari G. (2013). Prevention of diabetic foot ulcer. *International Journal of Preventive Medicine*.

[7] Cavanagh P. R., Lipsky B. A., Bradbury A. W., Botek G. (2005). Treatment for diabetic foot ulcers. *The Lancet*.

[8] Loveman E., Royle P., Waugh N. (2009). Specialist nurses in diabetes mellitus. *Cochrane Database of Systematic Reviews*.

[9] Ramachandran A. (2004). Specific problems of the diabetic foot in developing countries. *Diabetes/Metabolism Research and Reviews*.

[10] Kasiya M. M., Mang’anda G. D., Heyes S. (2017). The challenge of diabetic foot care: Review of the literature and experience at Queen Elizabeth Central Hospital in Blantyre, Malawi. *Malawi Medical Journal*.

[11] Boulton A. J., Armstrong D. G., Albert S. F. (2008). Comprehensive foot examination and risk assessment: a report of the task force of the foot care interest group of the american diabetes association, with endorsement by the american association of clinical endocrinologists. *Diabetes Care*.

[12] Jeffcoate W. J., Harding K. G. (2003). Diabetic foot ulcers. *The Lancet*.

[13] Bakker K., Apelqvist J., Schaper N. C. (2012). Practical guidelines on the management and prevention of the diabetic foot 2011. *Diabetes/Metabolism Research and Reviews*.

[14] American Diabetes Association (2013). Standards of medical care in diabetes. *Diabetes Care*.

[15] Alexiadou K., Doupis J. (2012). Management of diabetic foot ulcers. *Diabetes Therapy*.

[16] Schaper N. C., Van Netten J. J., Apelqvist J., Lipsky B. A., Bakker K., on behalf of the International Working Group on the Diabetic Foot (2016). Prevention and management of foot problems in diabetes: a summary guidance for daily practice 2015, based on the IWGDF guidance documents. *Diabetes/Metabolism Research and Reviews*.

[17] Clarke E. A. M., Tsubane M. (2008). The role of the podiatrist in managing the diabetic foot ulcer. *Wound Healing Southern Africa*.

[18] Boulton A. J., Vileikyte L., Ragnarson-Tennvall G., Apelqvist J. (2005). The global burden of diabetic foot disease. *The Lancet*.

[19] Karaca A., Enç N. (2005). Tip 2 Diabetes mellituslu hastalarin ayak komplikasyonlarinin belirlenmesinde hemsirenin rolü [The role of the nurse in recognition of foot complications in Type 2 diabetes mellitus]. *Endokrinoloji Forumu 3 (Diabet Bilimi Dergisi Ek’i)*.

[20] Waheida S., Elshemy M. B., Basal A. A. (2015). Effect of educational program about foot care on nurses' knowledge, practiceand outcomes for patients with diabetes. *IOSR Journal of Nursing and Health Science*.

[21] Mayfield J. A., Sugarman J. R. (2000). The use of the Semmes-Weinstein monofilament and other threshold tests for preventing foot ulceration and amputation in persons with diabetes. *The Journal of Family Practice*.

[22] Abbott C. A., Carrington A. L., Ashe H. (2002). The North-West Diabetes Foot Care Study: incidence of, and risk factors for, new diabetic foot ulceration in a community-based patient cohort. *Diabetic Medicine*.

[23] Lavery L. A., Peters E. J., Williams J. R., Murdoch D. P., Hudson A., Lavery D. C. (2008). Reevaluating the way we classify the diabetic foot: restructuring the diabetic foot risk classification system of the international working group on the diabetic foot. *Diabetes Care*.

[24] Seaman S. (2005). The role of the nurse specialist in the care of patients with diabetic foot ulcers. *Foot & Ankle International*.

[25] Yazdanpanah L., Nasiri M., Adarvishi S. (2015). Literature review on the management of diabetic foot ulcer. *World Journal of Diabetes*.

[26] Aydin K., Isildak M., Karakaya J., Gürlek A. (2010). Change in amputation predictors in diabetic foot disease: Effect of multidisciplinary approach. *Endocrine Journal*.

[27] Aalaa M., Malazy O. T., Sanjari M., Peimani M., Mohajeri-Tehrani M. (2012). Nurses’ role in diabetic foot prevention and care; a review. *Journal of Diabetes & Metabolic Disorders*.

[28] Viswanathan V., Madhavan S., Rajasekar S., Chamukuttan S., Ambady R. (2005). Amputation prevention initiative in South India: Positive impact of foot care education. *Diabetes Care*.

[29] Vijay V., Narasimham D. V. L., Seena R., Snehalatha C., Ramachandran A. (2000). Clinical profile of diabetic foot infections in south India - A retrospective study. *Diabetic Medicine*.

[30] Monami M., Zannoni S., Gaias M., Nreu B., Marchionni N., Mannucci E. (2015). Effects of a short educational program for the prevention of foot ulcers in high-risk patients: a randomized controlled trial. *International Journal of Endocrinology*.

[31] Global Report on Diabetes World Health Organization. http://apps.who.int/iris/bitstream/10665/204871/1/9789241565257_eng.pdf.

[32] Gershater M. A., Pilhammar E., Apelqvist J., Alm-Roijer C. (2011). Patient education for the prevention of diabetic foot ulcers: Interim analysis of a randomised controlled trial due to morbidity and mortality of participants. *European Diabetes Nursing*.

[33] Dorresteijn J. A. N., Kriegsman D. M., Assendelft W. J. J., Valk G. D. (2012). Patient education for preventing diabetic foot ulceration. *Cochrane Database of Systematic Reviews*.

[34] McCollum M., Hansen L. S., Lu L., Sullivan P. W. (2005). Gender differences in diabetes mellitus and effects on self-care activity. *Gender Medicine*.

[35] Shrivastava S. R., Shrivastava P. S., Ramasamy J. (2013). Role of self-care in management of diabetes mellitus. *Journal of Diabetes and Metabolic Disorders*.

[36] Stolt M., Suhonen R., Puukka P., Viitanen M., Voutilainen P., Leino-Kilpi H. (2015). Nurses’ knowledge of foot care in the context of home care: a cross-sectional correlational survey study. *Journal of Clinical Nursing*.

[37] Aalaa M., Sanjari M., Shahbazi S. (2017). Diabetic foot workshop: Improving technical and educational skills for nurses. *Medical Journal of The Islamic Republic of Iran*.

[38] Siminerio L. M., Funnell M. M., Peyrot M., Rubin R. R. (2007). US nurses' perceptions of their role in diabetes care: Results of the cross-national Diabetes Attitudes Wishes and Needs (DAWN) study. *The Diabetes Educator*.

[39] Kır Biçer E. Diyabetli Hastalarda Ayak Bakım Uygulamaları ve Öz Etkililiğin Değerlendirilmesi [Evaluation of Foot Care Practices and Self Efficacy for Patients with Diabetes].

[40] Moreira R. C., Sales C. A. (2010). The nursing care towards individuals with diabetic foot: a phenomenological focus. *Rev EscEnferm USP*.

[41] Ren M., Yang C., Lin D. Z. (2014). Effect of intensive nursing education on the prevention of diabetic foot ulceration among patients with high-risk diabetic foot: A follow-up analysis. *Diabetes Technology & Therapeutics*.

[42] Batkin D., Çetinkaya Ç. (2005). Diabetes mellitus hastalarinin ayak bakimi ve diabetik ayak hakkindaki bilgi, tutum ve davranislari [The knowledge, attitude and behaviours of the diabetic patients on diabetic foot and foot care]. *Saglik Bilimleri Dergisi*.

[43] Namwong T. Nursing Practice Guideline for Foot Care for Patients with Diabetes in Thailand.

[44] Çaparusagi Nese A., Ovayolu N. (2006). Diyabetik ayak bakim [Diabetic foot and its care]. *Atatürk Üniversitesi Hemsirelik Yüksekokulu Dergisi*.

[45] Aydoğan A. Hemşirelerin Diyabet ile ilgili Bilgi Düzeylerinin Tespiti [The Establishment of Knowledge Level of Nurses about Diabetes].

